# Community involvement in eye care: a health systems perspective

**Published:** 2022-09-20

**Authors:** Sumrana Yasmin, Hannah Faal, Thulasiraj Ravilla, GVS Murthy, BR Shamanna

**Affiliations:** Deputy Technical Director, Eye Health and URE: Sightsavers, Islamabad, Pakistan.; Adjunct Professor of International Eye Health: University of Calabar Teaching Hospital, Calabar, Nigeria.; Director-Operations: Aravind Eye Care System, Madurai, India.; Director: Indian Institute of Public Health, Hyderabad, India.; Professor: School of Medical Sciences, University of Hyderabad, India.

**Active community participation in the six pillars of the health systems strengthening framework is vital if we are to achieve universal access to eye care.**

There are 2.2 billion people around the world who have a visual impairment. Almost half of them have an eye condition which is either preventable or treatable, and is yet to be addressed.^1^ Of these 1.1 billion people who are still in need of eye care, 55% are women and girls and most live in low- and middle-income countries,^2^ where they have limited access to eye health services due to various socio-economic and cultural barriers, made worse by the COVID-19 pandemic.

**Figure:**
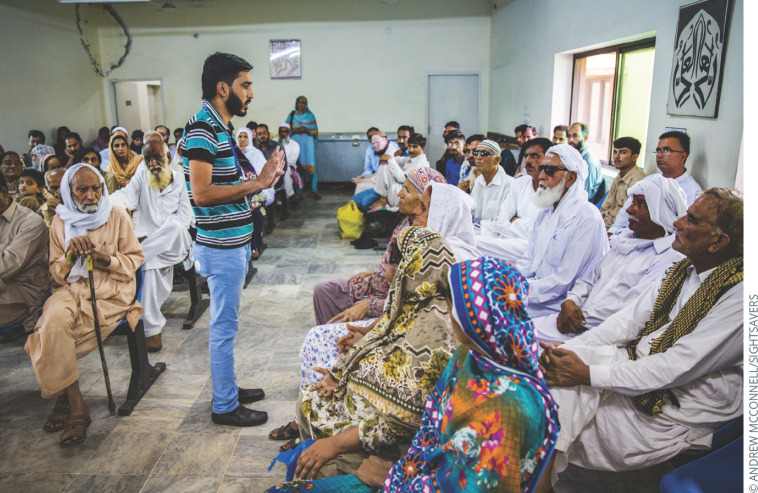
Engaging with communities is a vital part of extending eye care to all. pakistan

The **World Health Organization's World Report on Vision (2019)** and **The Lancet Global Eye Health Commission on Global Eye Health** highlight key priority areas for action that include the integration of eye care into universal health care (UHC) and delivering integrated people-centred eye care for all.^3^ Most of this can be managed and sustained through routine primary health care services (see previous online issue: **https://www.cehjournal.org/primary-eye-care/**).

Engaging with communities and empowering them to be part of this journey – right from the outset – is vital to maximise efforts to improve the delivery, acceptance, and uptake of eye care services.^4^ If we want to ensure that this engagement is effective and optimises eye care, we need to place communities in the driver's seat. This article describes what should be in place to make this possible, with reference to the six pillars of the health systems strengthening framework: service delivery, the health workforce, health information systems, essential medicines (here extended to include supplies), financing, and leadership/governance. Communities should be involved in all six pillars where possible, through active participation and engagement in planning and implementation.

## Service delivery

Communities, as well as local and national governments, must be included in the design and delivery of eye health programmes and interventions. It is more effective in the long term to use a participatory approach and to work in consultation with community leaders to ensure all voices are heard. Communities can be empowered to engage in delivering eye care, to support access to screening, treatment and surgery, and to strengthen referral pathways. It is just as important to listen to different groups in the community to ensure that their needs are being met and that any barriers to access are removed. This is vital to ensure equity of access to eye health services by different population groups, e.g., women, people with disabilities, and everyone regardless of their gender identity or sexual orientation.

## Health workforce

Health systems should be well equipped with competent staff members who have the skills and tools to provide eye care. There must be training and continuous support for different categories of health workers at community, primary, secondary, and tertiary levels of care. A competent and well-resourced eye health workforce that is integrated into the health system and accessible to communities in need will not only increase access to care but will also help to ensure a continuum of care for patients – from initial detection/screening to treatment, follow-up, and rehabilitation (if needed). As we take eye care to village and household levels, the community can play a critical role in providing volunteer workers that can help to mobilise patients or carry out simple screening, thereby supplementing the efforts of skilled health workers. Such a ‘community health system’ is informal, traditional, and familiar, which can support people to overcome barriers such as fear and distrust.

## Health information systems

National and provincial/regional eye health programmes must be represented within the national health information management system (HMIS) and integrated with other relevant databases. However, quantitative data alone doesn't capture the complete story. It is equally important to focus on human stories from and by the communities who are impacted by these programmes. A mechanism to bring the ‘human’ focus into information management needs to be considered at the community level. Data about resource availability and performance should not only be captured in the information system but also compared against the data on the eye care needs of the community. Such denominator-driven health information systems are essential if we are to know where we are in the journey towards universal health care.

## Supplies and technology

Addressing vision impairment requires strengthening the supply of medical products, spectacles, assistive technologies, and data management at all levels. Private sector providers, such as community pharmacies, can also play an important part if appropriately trained, regulated, and integrated with the health system. Since primary eye health care is a key strategy, we will need to ensure that supply chain challenges are addressed in areas that are difficult to reach (known as the ‘last mile’). This will have a lasting and sustainable impact on the affordability and quality of eye care, particularly for underserved communities and groups.

## Finance

Sustaining eye health services in low and/or middle-income countries is a major challenge, which makes it necessary to select and implement appropriate business models for public and private eye health facilities and systems as early as possible. Domestic financing and community-based financing mechanisms can help to build cost-effective, well-resourced, cross-sectoral delivery models for eye care and strengthen health system financing. Policy integration of this aspect is vital to ensure sustainability and coverage of essential eye health services across the population, so that we leave no one behind.

## Leadership and governance

High-level leadership is mostly engaged in advocating for the elimination of unaddressed vision impairment. However, in many contexts, community-level groups can be involved in advocacy and governance. These include education and rehabilitation groups, organisations of people with disabilities, and women's groups. These groups can hold eye care service providers to account, ensuring they are more responsive to local needs. To strengthen the health system through the development of comprehensive primary eye care, there is an urgent need for leadership to focus on enabling policies relating to investigations, treatment, and rehabilitation.

## Mobile communications technology

An aspect not covered in the health systems strengthening framework is communications technology, particularly as it relates to cellphones (also known as mobile phones) and how they are being used to support community mobilisation and eye care delivery. Other articles in this issue (**bit.ly/CEHJcommunity**), and in our recent issue on communication technology for eye care (**bit.ly/CEHJtech****)**, offer useful examples.

In conclusion, active community participation in the six pillars of the health systems strengthening framework, as well as community engagement in the implementation of policies, is vital if we want to achieve universal eye health coverage.
